# Cellular Uptake and Bioavailability of Tocotrienol-Rich Fraction in SIRT1-Inhibited Human Diploid Fibroblasts

**DOI:** 10.1038/s41598-018-28708-z

**Published:** 2018-07-11

**Authors:** Faizul Jaafar, Asmaa Abdullah, Suzana Makpol

**Affiliations:** Department of Biochemistry, Faculty of Medicine, Level 17, Preclinical Building, Universiti Kebangsaan Malaysia Medical Centre, Jalan Yaacob Latif, Bandar Tun Razak, Cheras, 56000 Kuala Lumpur Malaysia

## Abstract

Tocotrienol-rich fraction (TRF) is palm vitamin E that consists of tocopherol and tocotrienol. TRF is involved in important cellular regulation including delaying cellular senescence. A key regulator of cellular senescence, Sirtuin 1 (SIRT1) is involved in lipid metabolism. Thus, SIRT1 may regulate vitamin E transportation and bioavailability at cellular level. This study aimed to determine the role of SIRT1 on cellular uptake and bioavailability of TRF in human diploid fibroblasts (HDFs). *SIRT1* gene in young HDFs was silenced by small interference RNA (siRNA) while SIRT1 activity was inhibited by sirtinol. TRF treatment was given for 24 h before or after SIRT1 inhibition. Cellular concentration of TRF isomers was determined according to the time points of before and after TRF treatment at 0, 24, 48, 72 and 96 h. Our results showed that all tocotrienol isomers were significantly taken up by HDFs after 24 h of TRF treatment and decreased 24 h after TRF treatment was terminated but remained in the cell up to 72 h. The uptake of α-tocopherol, α-tocotrienol and β-tocotrienol was significantly higher in senescent cells as compared to young HDFs indicating higher requirement for vitamin E in senescent cells. Inhibition of *SIRT1* gene increased the uptake of all tocotrienol isomers but not α-tocopherol. However, SIRT1 inhibition at protein level decreased tocotrienol concentration. In conclusion, SIRT1 may regulate the cellular uptake and bioavailability of tocotrienol isomers in human diploid fibroblast cells while a similar regulation was not shown for α-tocopherol.

## Introduction

Vitamin E is an essential lipophilic antioxidant synthesized in plant particularly found in seed and oil. Natural vitamin E consists of 2 isomers, tocopherol and tocotrienol. Each isomer occurs in four different forms α-, β-, γ- and δ-. The difference between these two isomers depends on the isoprenoid chain in which tocopherol contains a saturated phytyl chain meanwhile tocotrienol contains an unsaturated geranyl chain. Vitamin E is widely known as lipid soluble antioxidant that protects lipid phase of circulating lipoprotein and cell membrane from oxidative damage^1^. Reduction of peroxyl radicals in lipid phase prevents oxidative damage-related diseases such as cardiovascular disease, degenerative disease and cancer^2^. Recent finding showed that vitamin E exerts its function in certain domain of the membrane which modulates the signalling pathway and gene regulation^3^.

Vitamin E is considered as a biologically important molecule that has dual roles of its function. The non-antioxidant roles of vitamin E has gained tremendous attention. It has been found to act as a signalling molecule in cell signalling pathway, and regulates gene expression, immune response, inflammation pathway and apoptosis^4^. Previously, vitamin E has been broadly studied, however the studies mainly focused on α-tocopherol isomer. Tocotrienol gradually garnered attention as it was found to exert better antioxidant activity and was shown to have different mechanism of action as compared to tocopherol^5^. The non-antioxidant activity of tocotrienol includes modulation of gene and protein expression in ageing, cancer and neurodegenerative diseases^6–8^. Previous studies conducted on vitamin E mostly showed the effect of single isomer, either of tocopherol or tocotrienol. However, a combination of vitamin E isomers, α-tocopherol and all forms of tocotrienol in palm tocotrienol-rich fraction (TRF) has been shown to exert better effect as compared to single isomer of vitamin E in improving endothelium-dependent relaxation during oxidative stress^9^.

Palm TRF is a natural mixture of vitamin E isomers that consist of 25% α-tocopherol and 75% tocotrienol (α, β, γ and δ). Palm TRF prevents cellular senescence of human skin diploid fibroblasts (HDFs) by elongating telomere length, regulating the expression of genes and proteins involved in signalling pathway, reducing damaged DNA and regulating cell cycle progression^10^. Furthermore, palm TRF is also involved in modulating gene expression of longevity protein, SIRT1. The expression of SIRT1 in young and senescent HDFs was upregulated with palm TRF treatment^11^.

The bioavailability of vitamin E in blood circulation has been studied widely. Among 8 forms of vitamin E, α-tocopherol is predominantly found in the blood plasma. Analysis of vitamin E in fasting human subjects showed the presence of α-tocopherol while none of other forms of vitamin E was present in the plasma^12^. Supplementation of α-tocopherol and tocotrienols significantly increased plasma α-tocopherol which reached the highest concentration peak after 8 hours of supplementation. Meanwhile, tocotrienols increased significantly in plasma after 2 hours and reached the highest concentration peak after 4 hours of supplementation. The concentration of α-tocopherol remains high in the plasma even after 24 hours of supplementation. Tocotrienols however was significantly reduced after 4 hours of supplementation and completely disappeared from plasma after 24 hours^13^. The availability of vitamin E in blood plasma is associated with the binding of vitamin E to lipoprotein molecules^14^. These findings showed that α-tocopherol has longer half-life which is 8 hours as compared to tocotrienols which is only 4 hours. Analysis of vitamin E distribution in lipoprotein showed that α-tocopherol is present in all lipoprotein molecules and triglyceride particle (TRP). α-Tocopherol was mostly found in very low density lipoprotein (VLDL), low density lipoprotein (LDL) and high density lipoprotein (HDL)^15^. Tocotrienols isomer however was found mainly in HDL. Tocotrienol was also found in LDL and TRP, however the tocotrienol isomer content in these two molecules was significantly lower as compared to in HDL molecule^12^.

At tissue level, vitamin E particularly α-tocopherol is widely distributed in most tissues. The presence of α-tocopherol is associated with the expression of LDL receptor which is expressed in most tissues^16^. α-Tocopherol is mainly found in hepatic tissues, skeletal muscle and adipose tissue. Among these tissues, adipose tissue which is rich of fat serves as vitamin E storage organ^17^. Tocotrienol isomers are found in selective tissues such as skin, liver, heart, lung and brain. Similar to tocopherol isomer, tocotrienol is highly found in adipose tissue which also serves for tocotrienol storage^18^. The presence of tocotrienol in selective tissues is explained by abundance of tocotrienol in HDL molecule. HDL receptors particularly scavenger receptor class B, type I (SR-B1), a membranous protein receptor was found to mediate selective uptake of α-tocopherol bound to HDL molecule^19^. SR-B1 protein is expressed in several tissues such as liver, brain, lung, skin and steroidogenic tissues^20^. Even though, the uptake of tocotrienol in certain tissues is associated with HDL receptor, the cellular uptake and bioavailability remains elusive. Even though the half-life of tocotrienol in plasma has been discovered, the half-life of tocotrienol at cellular level remains questionable. Thus, this study was conducted to determine the bioavailability of tocotrienol isomer in human skin fibroblast cells.

Previous studies have shown that SR-B1 protein was associated with SIRT1 protein^21^. SIRT1 is the longevity protein that has gained attention in aging research due to its role as a central regulator of aging process. SIRT1 belongs to NAD^+^-dependent deacetylase protein that catalyses the deacetylation process. The target protein of SIRT1 include p53, FOXO3a, NF-κB, PPAR-γ and c-Myc which are involved in cell proliferation, inflammation, stress response and apoptosis^22^. Furthermore, SIRT1 deacetylates proteins which are involved in the regulation of cellular metabolism particularly lipid metabolism and homeostasis. Knockdown of SIRT1 protein in mice decreased serum cholesterol and increased hepatic free fatty acids and cholesterol levels. Meanwhile overexpression of SIRT1 reduced total cholesterol in hepatic tissue. Influx and efflux of lipid particularly cholesterol is regulated by several transport proteins including SR-B1, cell membrane ATP binding cassette (ABC) transporters ABCA1 and ABCG1. Inhibition of SIRT1 through shRNA decreased the expression of SR-B1 and ABCA1^21^. Vitamin E is packed together with triacylglycerol, phospholipid and cholesterol in chylomicron which later taken up by the liver. Recent study showed that attenuation of SIRT1 activity in the liver increases lipogenesis and reduces the mobilization of lipid^23^. Thus, the aim of this study was to determine the role of SIRT1 on cellular uptake and bioavailability of tocotrienol isomer in human skin fibroblast cells.

## Results

### Optimum Dosage determination of TRF treatment

**Figure 1:**
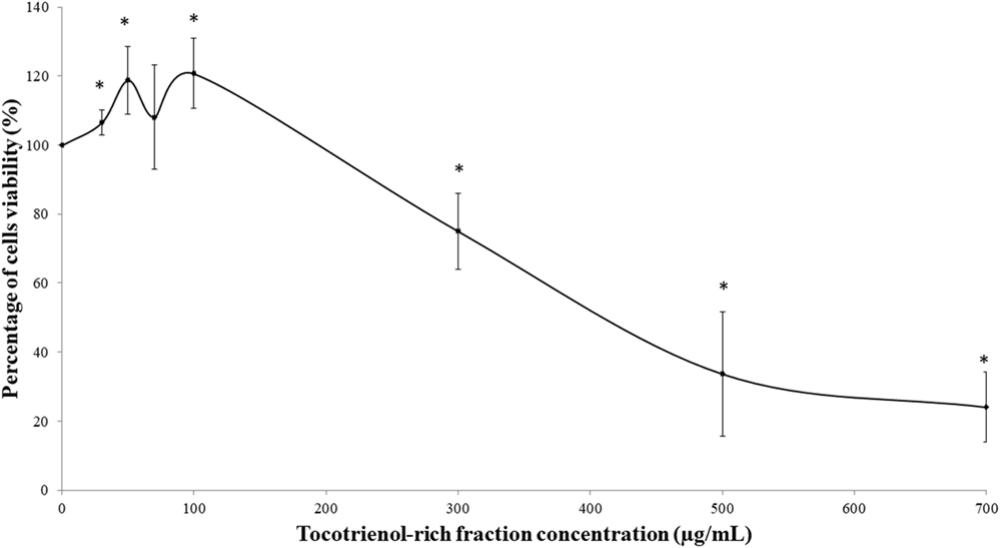
Effect of different dosages of tocotrienol-rich fraction (TRF) on the viability of human diploid fibroblast cells. Data represents mean ± SD of TRF concentration. *Significant different compared to hour 0 (p < 0.05).

The optimum TRF dosage was determined by viability assay. The screening of dosage was carried out by treating HDFs with several dosages of TRF; 0, 30, 50, 70, 100, 300, 500 and 700 µg/mL. TRF at 30, 50 and 100 µg/mL increased cell viability significantly as compared to untreated control (p < 0.05) (Fig. 1). TRF at 70 µg/mL did not cause a significant increase or decrease in the percentage of cell viable. Meanwhile, dosages of TRF at 300, 500 and 700 µg/mL attenuated the viability of HDFs significantly as compared to untreated HDFs. Thus, TRF at 50 µg/mL was selected as the optimum dosage used for subsequent experiment.

### Cellular bioavailability of TRF in young human diploid fibroblasts

**Figure 2:**
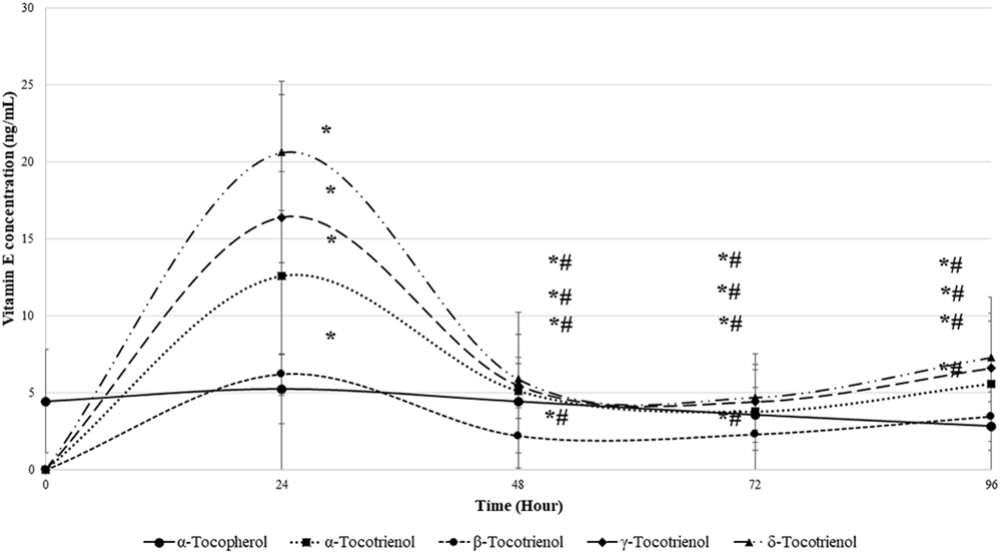
Bioavailability of tocotrienol-rich fraction (TRF) isomers in young human diploid fibroblast cells. Data represents mean ± SD of TRF concentration over time. *Significant different compared to hour 0 (p < 0.05), ^#^significant different compared to hour 24 (p < 0.05).

The level of all tocotrienol isomers in young HDFs was elevated significantly at hour 24 as compared to hour 0 (p < 0.05) (Fig. 2). However, the level of α-tocopherol isomer showed no significant increase or decrease as compared to HDFs at 0 h . After 24 h of TRF-containing medium removal (hour 48), the concentration of all tocotrienol isomers decreased significantly as compared to hour 24 (p < 0.05). However, the tocotrienol isomers was not removed completely from the cell and the concentration was maintained around 2–7 ng/mL until hour 96. The concentration of α-tocopherol however remain unchanged from hour 0 until hour 96.

### Effect of SIRT1 inhibition on cellular uptake of TRF in human diploid fibroblasts

**Figure 3:**
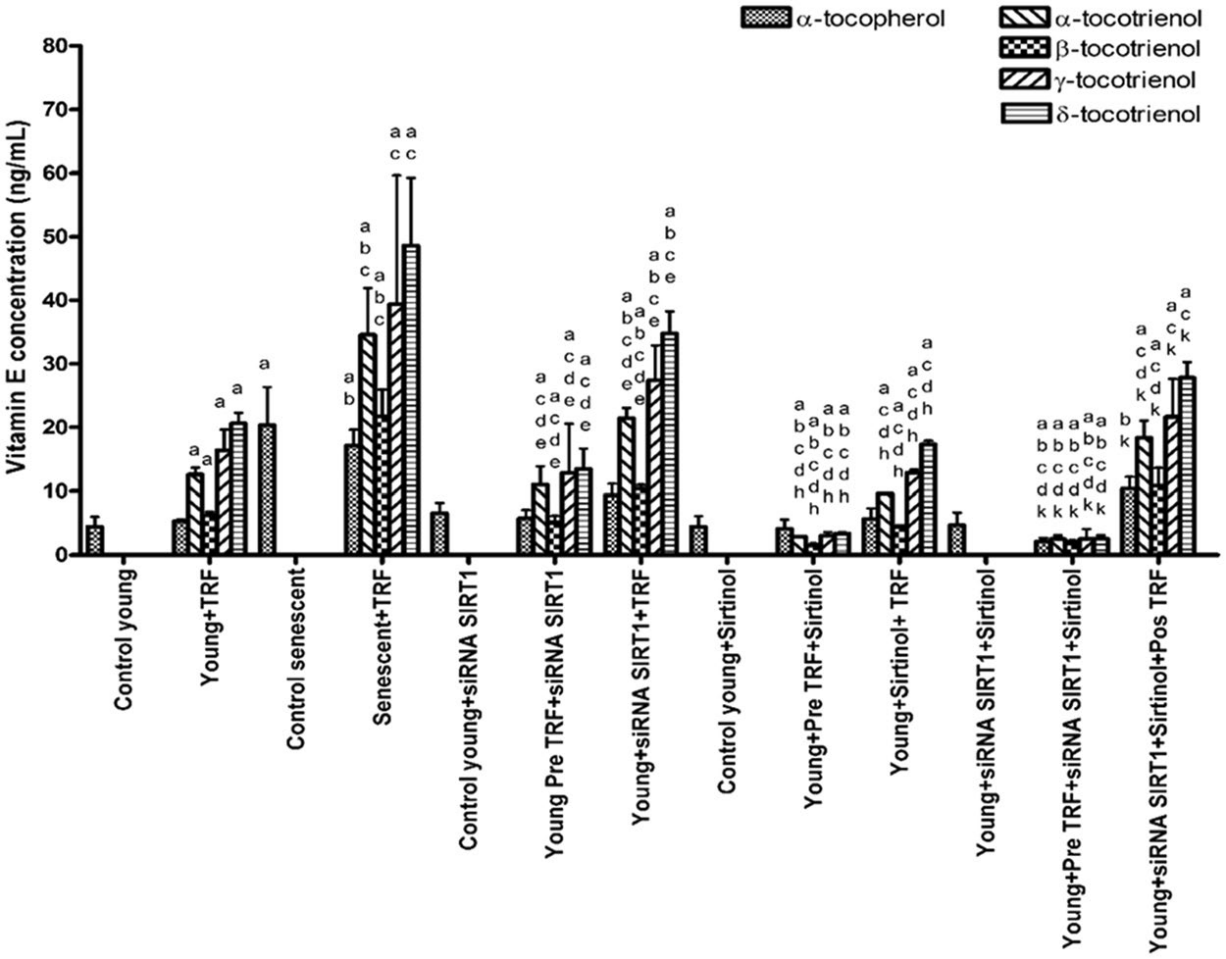
Effect of SIRT1 on vitamin E uptake of human diploid fibroblast cells. Each data represents mean ± SD of vitamin E concentration. ^a^Significant different compared to control young (p < 0.05), ^b^significant different compared to control young TRF-treated (p < 0.05), ^c^significant different compared to control senescent (p < 0.05), ^d^significant different compared to control senescent TRF-treated (p < 0.05), ^e^significant different compared to young with *si*RNA-*SIRT1* transfected (p < 0.05), ^h^significant different compared to young with sirtinol-treated (p < 0.05), ^k^significant different compared to young with *si*RNA-*SIRT1* transfected and sirtinol-treated (p < 0.05).

TRF treatment to young and senescent HDFs caused a significant increase of all tocotrienol isomers as compared to untreated young and senescent fibroblast cells (p < 0.05) (Fig. 3). The concentration of α-tocopherol, and α- and β-tocotrienol in TRF-treated senescent HDFs increased significantly as compared to young HDFs treated with TRF. In addition, the concentration of α-tocopherol in untreated senescent HDFs was significantly higher as compared to untreated young HDFs.

TRF treatment prior to silencing of *SIRT1* gene in young HDFs caused a significant increase of all tocotrienol isomers concentration as compared to untreated young HDFs and untreated young HDFs with silencing of *SIRT1* gene (p < 0.05). In addition, TRF treatment after silencing of *SIRT1* gene in young HDFs caused further increase of all tocotrienol isomers concentration as compared to untreated young HDFs, young HDFs treated with TRF alone and young HDFs with silencing of *SIRT1* gene (p < 0.05). However, no significant changes were observed on α-tocopherol concentration in all group of cells with *SIRT1* gene silencing.

TRF treatment prior and after inhibition of SIRT1 protein in young HDFs caused a significant increase of all tocotrienol isomers concentration compared to untreated young HDFs with SIRT1 protein inhibition (p < 0.05). However, concentration of all tocotrienol isomers in TRF-treated cells with treatment prior to SIRT1 inhibition was significantly lower as compared to young HDFs treated with TRF alone (p < 0.05).

TRF treatment prior and after the inhibition of SIRT1 at gene and protein levels increased the concentration of all tocotrienol isomers significantly as compared to the untreated group (p < 0.05). In addition, concentration of all tocotrienols isomers in TRF-treated group prior to SIRT1 inhibition at gene and protein levels was significantly lower as compared to TRF-treated young HDFs (p < 0.05).

### Effect of SIRT1 inhibition on cellular bioavailability of TRF isomers in fibroblast cells

**Figure 4:**
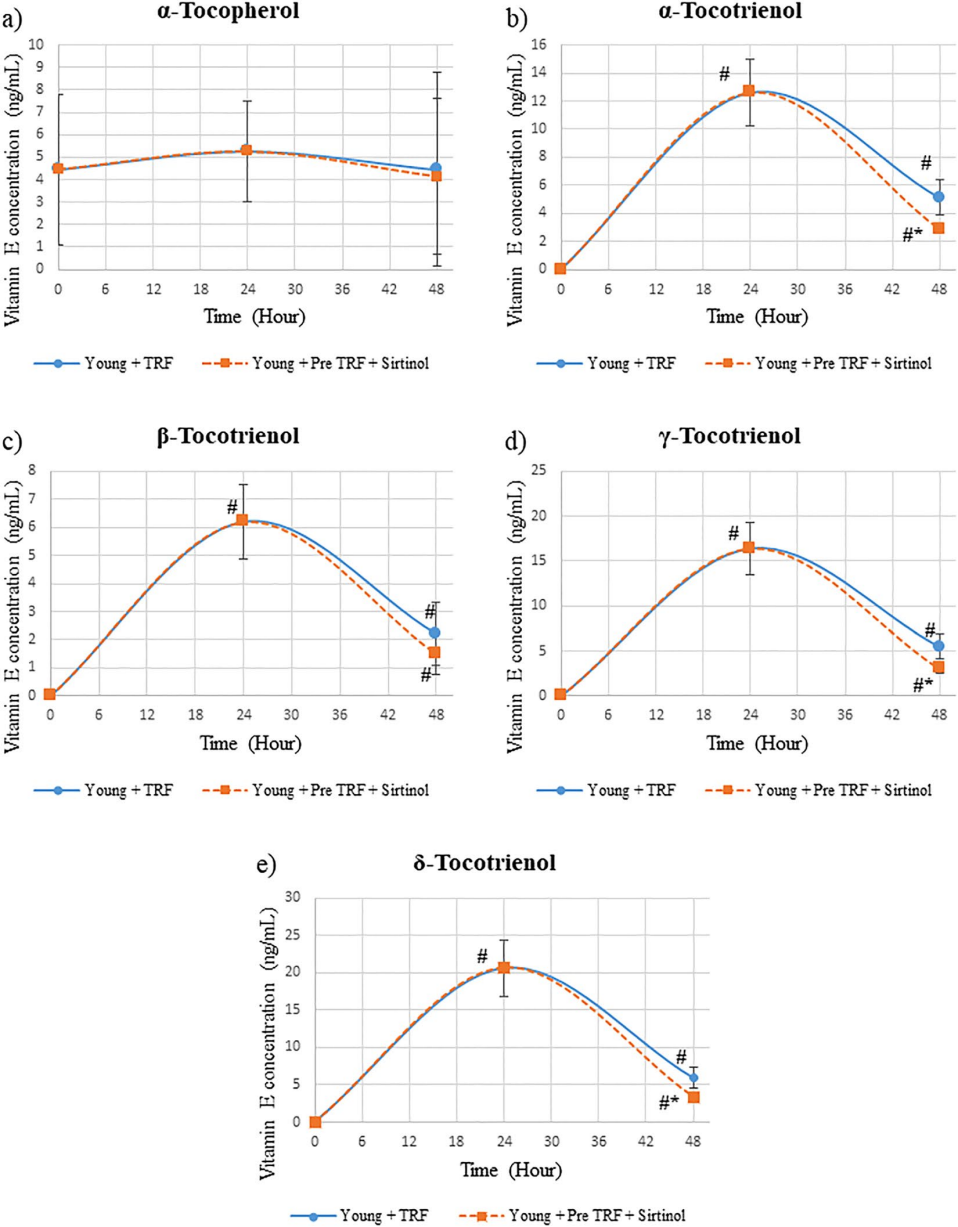
Concentration of tocotrienol-rich fraction (TRF) isomers over time in human diploid fibroblast cells. HDFs were treated with TRF before SIRT1 protein inhibition. Data represents mean ± SD of TRF concentration over time. *Significant different compared to young TRF-treated. ^#^Significant different compared to TRF treatment at 0 h.

Inhibition of SIRT1 protein after TRF treatment decreased the concentration of α-, γ- and δ-tocotrienol isomers significantly as compared to young HDFs treated with TRF alone (p < 0.05) (Fig. 4(b,d and e) (p < 0.05). Meanwhile, no significant reduction was observed in α-tocopherol and β-tocotrienol concentrations of HDFs with SIRT1 protein inhibition as compared to young HDFs treated with TRF alone (Fig. 4(a and c).

**Figure 5:**
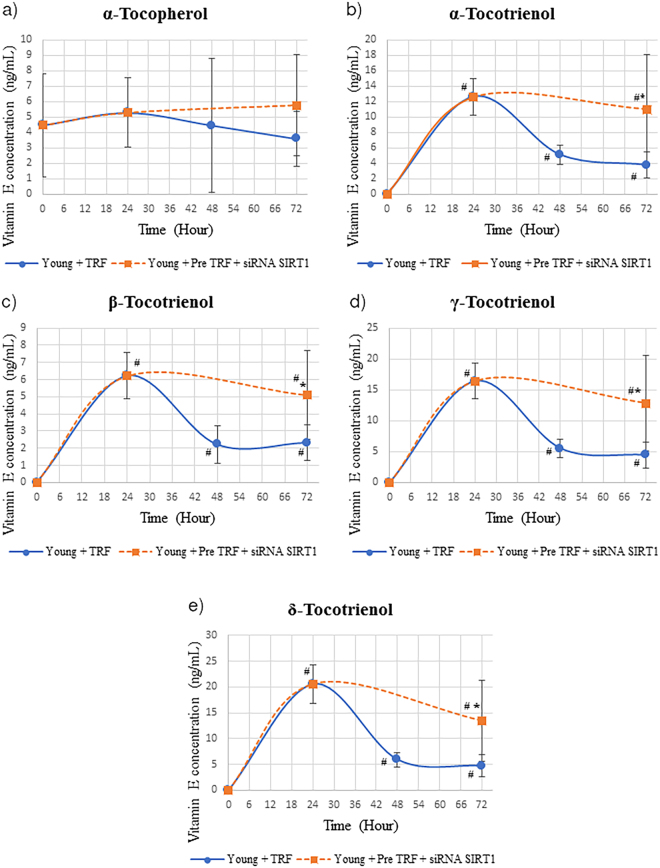
Concentration of tocotrienol-rich fraction (TRF) isomers over time in human diploid fibroblast cells. HDFs were treated with TRF before *SIRT1* gene inhibition. Data represents mean ± SD of TRF concentration over time. *Significant different compared to young TRF-treated. ^#^Significant different compared to TRF treatment at 0 h.

**Figure 6:**
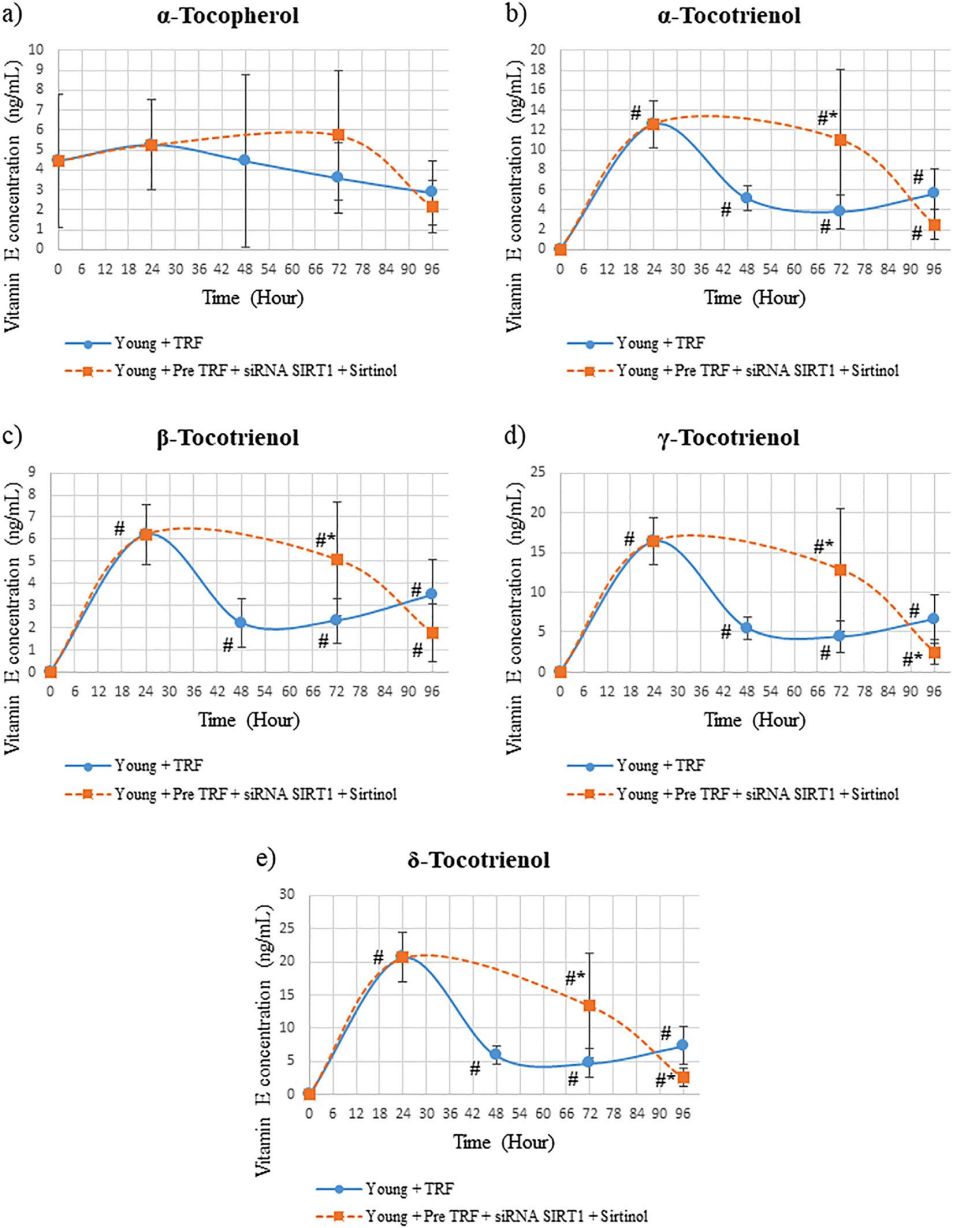
Concentration of tocotrienol-rich fraction (TRF) isomers over time in human diploid fibroblast cells. HDFs were treated with TRF before SIRT1 gene and protein inhibition. Data represents mean ± SD of TRF concentration over time. *Significant different compared to young TRF treated. ^#^Significant different compared to TRF treatment at 0 h.

Silencing of *SIRT1* gene in young HDFs after TRF treatment maintained the concentration of all tocotrienol isomers at hour 72 as compared to TRF concentration 24 h after TRF treatment (Fig. 5(b,c,d and e). The concentration of all tocotrienol isomers was also significantly higher in HDFs treated with TRF before silencing of *SIRT1* gene as compared to TRF-treated HDFs alone (p < 0.05). Meanwhile, no significant changes were shown in α-tocopherol concentration of young HDFs with *SIRT1* gene silencing as compared to young HDFs treated with TRF alone (Fig. 5(a)). For HDFs with inhibition of both *SIRT1* gene and SIRT1 protein, all tocotrienol isomers concentration was maintained significantly higher after *SIRT1* gene inhibition but reduced when SIRT1 protein was inhibited (p < 0.05) (Fig. 6(b,c,d and e). Comparison with TRF-treated HDFs alone showed that HDFs with *SIRT1* gene inhibition had higher tocotrienol isomers (p < 0.05). No significant reduction or elevation was observed in α-tocopherol concentration with inhibition of both SIRT1 gene and protein (Fig. 6(a)).

## Discussion

Vitamin E is an essential micronutrient for neuroprotection and reproduction which has been described since 1922^24^. Deficiency of vitamin E leads to several diseases such as neurodegenerative disease^25^. As vitamin E is only synthesized in plant, thus it is required in human diet. Vitamin E from the diet is absorbed in the intestine followed by secretion into lymphatic system. Subsequently, it enters the blood circulation system and then transported to the liver or extrahepatic tissues^26^. The uptake of vitamin E by different organs and tissues has been described in previous studies^27^. Among the 8 forms of vitamin E, the uptake of α-tocopherol has been studied vastly as compared to the tocotrienol isomer. In addition, α-tocopherol is predominantly found in most organ and tissues^28^. However, the uptake of tocotrienol isomer into the cells and its mechanism remain uncertain.

The molecular mechanism of vitamin E transport has been described previously which was referring to the transportation of α-tocopherol. Similar to lipid, transportation of vitamin E into the cell requires membrane transport proteins such as lipoprotein receptors^29^. Upon entering the cells through endocytosis, vitamin E is transferred out from chylomicron and binds to intracellular α-tocopherol transport protein (α-TTP). Then, the vitamin E is transferred to lipoprotein including LDL, VLDL and HDL^30^. There are three properties required for efficient binding to α-TTP which include the presence of (i) three methyl group at chromanol head, (ii) one free hydroxyl group and (iii). phytyl side chain. Hereby, α-tocopherol has 100% affinity to bind with α-TTP followed by β-tocopherol, α-tocotrienol, γ-tocopherol, δ-tocopherol and β-tocotrienol, γ-tocotrienol and δ-tocotrienol^31^. The binding of vitamin E to α-TTP prevents this compound from undergo β-oxidation of lipid metabolism^32^. Besides, α-TTP is also involved in the translocation of vitamin E to plasma membrane for exocytosis or efflux process which is mediated by membrane transport proteins. A recent finding showed that supplementation of vitamin E mixture results in a significantly increased tocotrienols tissue concentrations in various organs such as blood, skin, adipose, brain, cardiac muscle and liver^18^ indicating the presence of another mechanism of intracellular transport for tocotrienol isomer which is α-TTP independent.

Previously, findings from *in-vitro* study of enterocytes cells showed that all forms of vitamin E were equally taken up by the cells^15^. In the present study, we found that all tocotrienol forms were being taken up by fibroblast cells and the concentration of δ-tocotrienol was the highest followed by γ-tocotrienol, α-tocotrienol and β-tocotrienol. In contrast with other study, erythrocytes cells selectively taken up γ-tocotrienol followed by other forms of vitamin E^33^. Previous study reported that rats supplemented with vitamin E mixture which contains α-tocopherol, γ-tocopherol, α-tocotrienol and γ-tocotrienol extracted from palm oil showed higher uptake of α-tocotrienol followed by γ-tocotrienol in the skin^34^. Meanwhile, brain cells also showed different result as α-tocotrienol form is the major form found in this cells^18^. Hepatocyte cells showed that α-tocopherol is the major form of vitamin E taken up by this cells^28^. In this study we found that α-tocopherol was detected as early as at 0 hour whereby the cells had not been treated with TRF. HDFs were cultured in normal DMEM medium (without vitamin E) and supplied with 10% bovine serum. Previous study has shown the presence of α-tocopherol approximately 0.3 nmol/L in normal DMEM with 10% bovine serum^35^. Thus, this explains the presence of α-tocopherol in HDFs even though without TRF treatment. However, the findings of this study showed that α-tocopherol was not taken up by HDFs in TRF-treated cells and its level remained constant throughout the experiment. As various findings were shown from previous studies, we postulated that each type of cells has its own preferences and selectivity towards vitamin E uptake.

Vitamin E uptake is associated with several membrane transport proteins. At the intestine brush border, vitamin E uptake into enterocytes is mediated by Niemann-Pick C1-like 1 (NPC1L1) transporter, multi ligand HDL receptor, Scavenger receptor class B type I (SR-B1)^36^ and CD36 molecule (CD36)^29^. Meanwhile, in hepatic tissue, vitamin E uptake is mediated by LDL receptor, SR-B1 and CD36^37^. All of these membrane proteins are also expressed in other tissues. Previous studies have shown that the uptake of α-tocopherol occurs in most extrahepatic tissues which is associated with LDL receptor^38^ and SR-B1^39^. However an interesting finding was reported which showed that tocotrienol is selectively taken up by certain organs and tissues^18^. Similar to tocopherol isomer, tocotrienol was also found in tissues with high content of adipose such as the skin. The uptake of tocotrienol into tissues or cells is associated with HDL receptor particularly SR-B1^40^. This SR-B1 receptor is primarily expressed in the liver^41^, steroidogenic tissues and several non- steroidogenic tissues^42^ such as skin, thus explaining the reason why tocotrienol is only present in certain tissues and organ. In the present study, we found that HDFs selectively taken up the tocotrienol isomer. Previous study has shown cultured HDFs and human arterial smooth muscle cell expressed and have specific receptor for HDL, SR-B1 which possess high affinity binding for HDL^43,44^. Thus, we postulated that the selective uptake of tocotrienol in HDFs is associated SR-B1 receptor.

In this study, we found that the uptake of α-tocopherol and tocotrienol forms was increased in senescent HDFs as compared to young HDFs. Previously, study on human showed that uptake of tocotrienol into the plasma in older people increased as compared to younger people suggesting the need of tocotrienol in the elderly^45^. As we aged, the level of oxidant is high which requires more antioxidant to prevent oxidation of macromolecules. Thus, we postulated that increased uptake of α-tocopherol and tocotrienols in senescent HDFs is associated with increased expression of membrane proteins which are involved in vitamin E transport into the cells. Furthermore, these membrane transport proteins are suggested to be regulated by the proteins involved in senescence process. SIRT1 a key longevity protein was found to regulate numerous metabolic pathways which protect the cells from oxidative damage subsequently delayed the senescence process and prevent uncontrolled cells cycle progression^46^. The NAD^+^dependent of SIRT1 activity links this protein to the metabolic state particularly on lipid metabolism^47^. In response to nutritional and hormonal signal, SIRT1 has been shown to inhibit lipogenesis thus prevents lipid storage in the form of triglycerides and induce β-oxidation of fatty acid for degradation of lipid molecules^48^. Previous studies also showed that SIRT1 is involved in lipid uptake and transportation into the cells. Similar to vitamin E, transportation of lipid involved membrane transport proteins, LDL receptor, SR-B1, NPC1L1 and CD36. Lack of SIRT1 in heterozygous knockout (SIRT1^+/−^) increased the uptake of lipid into liver tissue and reduced the fat export^23^. SIRT1 expression in senescent cells has been reported to be significantly lower as compared to young HDFs^11^. Thus, we postulated that low expression of SIRT1 in senescent HDFs induced membrane transports protein to take up more vitamin E.

We extend our experiment by imitating senescent HDFs via inhibition of SIRT1 expression at mRNA or protein level and both at mRNA and protein levels in young HDFs. Our findings showed that the uptake of TRF was higher in young HDFs treated with TRF after *SIRT1* gene was inhibited. This observation was similar to senescent cells which showed increased uptake of all forms of vitamin E with low expression of SIRT1. However, inhibition of SIRT1 protein did not increase the uptake of all forms of vitamin E in TRF. Meanwhile, inhibition of SIRT1expression at both mRNA and protein levels increased the uptake of α-tocopherol only. Thus, we suggested that low expression of *SIRT1* mRNA stimulated the membrane transport protein to take up all forms of tocotrienol rapidly while inhibition of SIRT1 protein induced the slow uptake of tocotrienols or induced the secretion of tocotrienols out of the cell through ATP-binding cassette transporter (ABCA1). We also found that the concentration of all forms of tocotrienol was decreased in cells treated with TRF before SIRT1 protein inhibition as compared to cells treated with TRF after SIRT1 protein inhibition. A similar result was observed when TRF treatment was given before SIRT1 expression was inhibited at both mRNA and protein levels. As the concentration of vitamin E was measured after the experiment was completed, tocotrienol may be secreted out rapidly due to the inhibition of SIRT1 which further induced rapid secretion mediated by ABCA1. Furthermore, inhibition of SIRT1 may prevent the retention of vitamin E by intracellular transport protein which is associated with the cellular bioavailability of tocopherol and tocotrienol isomers.

Vitamin E homeostasis in the body is tightly maintained to prevent toxicity to the cells, tissues and subsequently to the organs. Maintaining vitamin E bioavailability in the cells involved influx and efflux processes which is mediated by membrane transport proteins^49^. This homeostasis process also involved intracellular transport proteins^49^. The bioavailability of α-tocopherol has been studied more extensively as compared to tocotrienol^50^. Previously, the study on vitamin E bioavailability mainly focused on the bioavailability in the blood plasma. Upon supplementation with vitamin E mixture, each of vitamin E form is taken up into blood circulation. The level of tocotrienols in the blood reaches the highest peak after 4 hours of supplementation. However, after 24 hours, tocotrienol completely disappear from the plasma. Meanwhile, tocopherol isomer particularly α-tocopherol reaches the highest peak after 8 hours and remained high even after 24 hours of vitamin E supplementation^12,13^. As mentioned earlier, a postprandial vitamin E supplementation results in α-tocopherol found in most of the tissues while tocotrienol was found only in selective tissues. And the bioavailability of these two isomers of vitamin E in the tissues particularly in cells remain elusive. In this study, the cellular bioavailability of vitamin E is defined as the level of vitamin E in an individual cell for a certain period of time^51^.

Different uptake rate of vitamin E forms in the liver cells is associated with intracellular transport protein known as α-TTP^52^. In the liver cells, vitamin E is transferred from chylomicron into the cells by binding to α-TTP. Previous study on hepatoma HepG2 cells showed that α-TTP was localized in late endosomes or lysosome^53^. α-TTP acquired the endocytosed vitamin E and translocated the vitamin E to the plasma membrane. While, ABCA1 transport protein mediated the secretion of vitamin E out of the cells and bind to lipoprotein. This transport protein is highly expressed in apical membrane of intestine and liver cells which mediates secretion of vitamin E into the lymph and blood circulation respectively^26^. Besides α-TTP, there are other transport proteins found to be involved in intracellular transportation of vitamin E such as cytosolic tocopherol associated protein (TAP)^54^ and tocopherol binding protein (TBP)^55^. These transport proteins have high affinity toward α-tocopherol compared to other forms of vitamin E.

Free vitamin E in the liver cells will subsequently be metabolized through β-oxidation^56^ thus reducing the cellular bioavailability of vitamin E in the liver cells which is the major organ for vitamin E metabolism. Previous study showed that α-TTP was also expressed in extrahepatic tissues such as kidney, brain, spleen, lung and skin^57,58^. In the present study, we found that the level of tocotrienol in young HDFs was increased and reached the highest peak after 24 hours of TRF supplementation and significantly decreased after 24 hours of TRF withdrawal and remained constant at concentration 2–7 ng/mL up to 72 hours. Cellular bioavailability of tocotrienol in HDFs up to 72 hours may involve tightly regulated of tocotrienol homeostasis. Thus, we suggested that the cellular bioavailability of tocotrienol in HDFs may involve SR-B1 for influx of tocotrienol and ABCA1 for efflux of tocotrienol. Meanwhile the retention of tocotrienol in HDFs is suggested to be dependent or independent of known intracellular transport protein of vitamin E. Further study is required to determine the other intracellular transport protein specific for tocotrienols.

In the present study, pre-treatment of TRF and inhibition of SIRT1 expression at mRNA or protein levels results in different vitamin E concentration in the cell. Upon TRF treatment, the level of tocotrienol was increased and reached the highest peak after 24 hours. The withdrawal of TRF followed by SIRT1 inhibition with sirtinol increased the secretion of tocotrienols. Meanwhile, the withdrawal of TRF followed by *SIRT1* gene inhibition prevents the secretion of tocotrienol. Thus, we extend our experiment to determine the cellular bioavailability of vitamin E upon SIRT1 inhibition at both mRNA and protein levels in young HDFs. Our findings showed that with the withdrawal of TRF and SIRT1 inhibition at both mRNA and protein levels, the level of all tocotrienol forms remained high after SIRT1 mRNA inhibition but was rapidly secreted out upon inhibition of SIRT1 at protein level. As discussed above the influx and efflux of vitamin E is mediated by SR-B1 and ABCA1 respectively. From this finding, we suggested that the cellular bioavailability of vitamin E particularly tocotrienol in HDFs is associated with the expression of SIRT1. While the retention of α-tocopherol in HDFs may involve α-TTP in maintaining its concentration in the cell throughout the study.

In summary, HDFs selectively taken up tocotrienol isomers compared to α-tocopherol. Senescent cells may require higher concentration of vitamin E as compared to young cells. We demonstrated that SIRT1 may regulate the uptake and bioavailability of tocotrienol isomers in HDFs.

## Methods

### Cell culture

Primary HDFs were derived from the foreskin of three boys’ donor aged between 8 to 12 years old after circumcision with parents’ informed consent. The collection of human tissue sample conformed to the Guidelines for Ethical Review of Clinical Research or Research Involving Human Subjects 2014, outlined by The University Kebangsaan Malaysia (UKM) and was approved by The Universiti Kebangsaan Malaysia Ethical Committee (Project code: FF-249-2012). The foreskins were washed aseptically for several times with 75% alcohol and phosphate-buffered saline (PBS) with 1% antibiotic-antimycotic solution (PAA, Austria). The fibroblast cell was extracted out by digesting the pure dermis in collagenase 0.03% type I solution (Worthington Biochemical Corporation, USA) for 6–12 h in an incubator shaker at 37 °C. Extracted fibroblast cells were then rinsed with PBS for several times and cultured in Dulbecco’s Eagle’s Medium (DMEM) (Thermo Fisher Scientific, USA) with 10% fetal bovine serum (FBS) (PAA, Austria) and 1% antibiotic-antimycotic solution at 37 °C in a 5% CO_2_ humidified incubator as previously described^10^. When cells confluence reached 80–90%, serial passaging was carried out whereby cells were harvested using accutase (PAA, Austria) and subcultured or culture-expanded in a new T75 culture flask (Nunc, Denmark) with expansion degree 1:4. Cells were cultured until senescence by monitoring the number of population doublings (PDs). The cells were used at passage 6 (young cells with PDs < 12) and passage 30 (senescent cells with PDs > 55) in subsequent experiment.

### ***SIRT1*gene silencing**

Young HDFs (passage 6) were transfected with 2 µM silent information RNA (siRNA) *SIRT1* (DharmaFECT, Thermo Fisher Scientific, USA) as per manufacturer’s protocol. The transfection process was carried out in serum free medium (DMEM without serum and antibiotic-antimycotic solution) and transfection reagent 1 (Thermo Fisher Scientific, USA) by 48 h incubation in a 5% CO_2_ humidified incubator at 37 °C.

### Inhibition of SIRT1 Activity

SIRT1 activity was inhibited by treating the cells with 60 µM sirtinol (Enzo Life Science, USA)^59^. Cells were initially washed with PBS and treated with sirtinol for 24 h in a 5% CO_2_ humidified incubator at 37 °C. Inhibition of SIRT1 at gene and protein levels was carried out in stages whereby the gene is silenced first followed by protein inhibition.

### Tocotrienol-Rich Fraction Treatment

TRF Gold Tri E 50 (Golden Hope Bioganic Sdn Bhd, Malaysia) was used in this study. The vitamin E isomers in TRF consist of 22.8% α-tocopherol, 26.06% α-tocotrienol, 3.65% β-tocotrienol, 31.35% γ-tocotrienol and 16.1% δ-tocotrienol. Stock solution of TRF (1 g/mL) was freshly prepared in 100% ethanol in 1:1 ratio and kept at −20 °C for not more than one month as previously described^10^. 45 µL of TRF stock solution were incubated with 60 µL FBS at 37 °C for overnight. Then, TRF stock solution was diluted to 150 mg/mL by adding 90 µL of culture medium and 105 µL of 100% ethanol. TRF stock solution was further diluted to 50 mg/mL by adding 600 µL of mixture of culture medium with 100% ethanol with ratio 1:1 (50% ethanol). Subsequently, the TRF stock solution was diluted to 1 mg/mL with culture medium only. Finally, from this concentration, the TRF stock solution was diluted to designated concentration with culture medium only. The TRF treatment was perform by replacing culture medium with TRF-containing medium and incubated in a 5% CO_2_ humidified incubator at 37 °C for 24 h.

### Viability assay

HDFs cells were seeded into 96 wells plate with density ~2 × 10^3^ cells/well in DMEM medium (Thermo Fisher Scientific, USA) with 10% fetal bovine serum (FBS) (PAA, Austria) and 1% antibiotic-antimycotic solution. Cells were incubated in a 5% CO_2_ humidified incubator at 37 °C for 24 h. The culture medium of fibroblast cells was then replaced with the new medium containing different dosages of TRF (0, 30, 50, 70, 100, 300, 500 and 700 µg/mL) and incubated in a 5% CO_2_ humidified incubator at 37 °C for another 24 h. After 24 h of TRF treatment, TRF-containing medium was replaced with normal medium containing CellTiter96* Aquous Non-Radioactive Cell Proliferation Assay solution as per manufacturer’s protocol. HDFs cells were incubated in a 5% CO_2_ humidified incubator at 37 °C for 2 h. Colour changes of the assay solution was measured by measuring the absorbance using spectrophotometer ELISA VersaMax (Molecular Device, USA).

### Determination of cellular bioavailability of vitamin E isomers of TRF in young HDFs

HDFs cells were seeded into 100 mm culture dish plate with cells density ~2 × 10^6^ cells/dish in DMEM medium (Thermo Fisher Scientific, USA) with 10% fetal bovine serum (FBS) (PAA, Austria) and 1% antibiotic-antimycotic solution. After incubation in a 5% CO_2_ humidified incubator at 37 °C for 24 h, the culture medium was replaced with the new medium containing 50 µg/mL of TRF. Then, the cells were incubated with TRF in a 5% CO_2_ humidified incubator at 37 °C for another 24 h. Determination of cellular bioavailability of vitamin E isomers was carried out at different time point: 24 h (after 24 h TRF treatment), 48 h (after 24 h TRF-containing medium was replaced with normal medium), 72 h (after 48 h TRF-containing medium was replaced with normal medium) and 96 h (after 72 h TRF-containing medium was replaced with normal medium) Upon completion, the cells were harvested for measurement of vitamin E isomers.

### Determination of cellular uptake of vitamin E isomers of TRF in SIRT1 inhibited HDFs

HDFs cells were seeded into 100 mm culture dish plate with cells density ~2 × 10^6^ cells/dish in DMEM medium (Thermo Fisher Scientific, USA) with 10% fetal bovine serum (FBS) (PAA, Austria) and 1% antibiotic-antimycotic solution. After 24 h, the cells were either treated with TRF (50 µg/mL) first for 24 h or underwent SIRT1 inhibition with *SIRT1* siRNA transfection for 48 h and sirtinol for 24 h and vice versa. Upon completion, the cells were harvested for measurement of vitamin E isomers.

### Determination of cellular bioavailability of vitamin E isomers of TRF in SIRT1 inhibited HDFs

HDFs cells were seeded into 100 mm culture dish plate with cells density ~2 × 10^6^ cells/dish in DMEM media (Thermo Fisher Scientific, USA) with 10% fetal bovine serum (FBS) (PAA, Austria) and 1% antibiotic-antimycotic solution. After 24 h of incubation, the cells were treated with TRF (50 µg/mL) for another 24 h. Then, the TRF-containing medium was removed out and the cells were treated with *SIRT1* siRNA transfection for 48 h and sirtinol for 24 h. Determination of cellular bioavailability of vitamin E isomers was carried out upon the completion of treatments at different time point: 48 h or 72 h or 96 h.

### Measurement of vitamin E isomers in HDFs using High performance liquid chromatography (HPLC)

The vitamin E in HDFs cells was extracted out by the following procedure as previously described with modification to allow vitamin E extraction from the cells^60^. The HDFs cells in culture dish were washed with PBS solution and harvested with accutase (PAA, Austria). HDFs cells were collected and counted. Subsequently, the cells were washed with cold PBS three times and suspended in DMEM medium followed by the addition of butylated hydroxytoluene (BHT) solution. The HDFs cells were incubated in ice for 10 mins. Then, cold ethanol solution was added and cells were sonicated at wavelength 200 nm for 40 secs to lyse the cells membrane. Hexane solution was added into cell lysate and centrifuged at 50000 rpm and 4 °C for 30 mins. Supernatant was transferred into new tube and dried up in vacuum concentrator centrifuge for 45 mins. After that, 30 µL of hexane was added to dissolve the vitamin E and then stored in −80 for further analysis.

HPLC LC-10AT system was used which consist of SCL-10A system controller and SIL-10A auto injector with cooler (Shimadzu, Japan). The stationary phase was silica normal phase column and mobile phase was a mixture of hexane:isopropanol (99:1). The flow rate was set at 1.5 mL/min and peaks were detected with excitation wavelength at 294 nm and emission wavelength at 300 nm. Peaks were integrated with LC workstation VP class software (Shimadzu, Japan). Pure TRF was used as standard curve and prepared from 2 µg/mL to 10 µg/mL with R^2^ > 0.990. Sample peaks obtained were compared with the standard. The total vitamin E concentration of α-tocopherol, α-tocotrienol, β-tocotrienol, γ-tocotrienol and δ-tocotrienol were calculated by multiplied the concentration of area under curve (AUC) with diluent constant, internal constant and divided with number of cells for normalisation. The formula for total vitamin E concentration calculation is shown as follows:

$$\begin{array}{c}{\rm{TRF}}\,{\rm{isomer}}\,{\rm{concentration}}\,\,({\rm{ng}}\,{\rm{per}}\,{\rm{mL}}\,{\rm{per}}\,{\rm{million}}\,{\rm{cells}})\\ \,=(\frac{{\rm{Isomer}}\,{\rm{concentration}}\times 10\,({\rm{Dilution}}\,{\rm{constant}})\times 0.741\,({\rm{Constant}})\times 0.001\,\times 0.5}{(\frac{{\rm{Number}}\,{\rm{of}}\,{\rm{cells}}\,{\rm{per}}\,{\rm{mL}}}{1000000})}\,)\,\times 1000\end{array}$$

### Statistical analysis

The sample data was obtained from three experiments  in duplicate of three biological skin samples. The Kruskal-Wallis test or One-way ANOVA on ranks was used to obtain significant different (p < 0.05) using SPSS software version 21.0. Data presented represents mean ± standard deviation (SD).
